# Regulation of NMDA receptor interactions with the actin cytoskeleton in dendritic spine development

**DOI:** 10.3389/fncel.2026.1813072

**Published:** 2026-05-29

**Authors:** Jarrett Lloyd, Karen Litwa

**Affiliations:** Department of Anatomy and Cell Biology, Brody School of Medicine, East Carolina Diabetes and Obesity Institute, East Carolina University, Greenville, NC, United States

**Keywords:** actin, dendritic spine, NMDA receptor, RhoGTPase, synapse

## Abstract

In the central nervous system, the majority of excitatory synapses exist on small actin-enriched structures protruding from dendrites, known as dendritic spines. Actin cytoskeletal rearrangements drive dynamic changes in spine shape, size, and density depending on developmental stage and synaptic activity. Spines initially emerge from the dendritic shaft as dynamic protrusions known as filipodia-like spines, which serve as precursors to mature dendritic spines. The formation and maturation of dendritic spines facilitate information transfer in neural circuits, underlying cognitive processes, such as learning and memory formation, and altered spine development results in neurodevelopmental disorders. Within dendritic spines, signaling events are regulated by many synaptic receptors such as the ionotropic N-methyl-D-aspartate (NMDA) and α-amino-3-hydroxy-5-methyl-4-isoxazolepropionic acid (AMPA) receptors. However, only NMDA receptors are enriched in both immature filopodia-like spine precursors and mature dendritic spines, with AMPA receptors expressed later in development and coinciding with spine maturation. During embryonic development, most NMDA receptors contain GluN2B subunits, in contrast to mature synapses which predominantly contain GluN2A subunits. While many actin regulatory proteins, such as α-actinin-2, interact with NMDA receptor subunits, it remains unclear whether GluN2B vs. GluN2A-containing receptors exhibit differences in their preferential protein interactions that underly dendritic spine development. The following review highlights how preferential interactions between specific GluN2 isoforms and actin cytoskeletal regulators underlie synaptic development by balancing dynamic events of synaptic plasticity with competing events of synaptic strengthening and consolidation. We discuss how alterations in the expression of GluN2 isoforms and/or mutations that disrupt actin interactions contribute to neurological disorders, both developmental and degenerative.

## Introduction to NMDA receptor structure and function

NMDA receptors (NMDARs) assemble as hetero-tetramers of GluN1 and GluN2 subunits to form transmembrane ion channel pores (Traynelis et al., 2010). GluN1 is found in all NMDA receptor complexes and plays an important regulatory role in NMDA receptor function through its binding to the NMDA receptor co-agonist, D-serine or glycine, while GluN2 subunits bind to the excitatory neurotransmitter glutamate and uniquely regulate the electrophysiological properties of the ion channel (Traynelis et al., 2010). Assembly of the NMDA receptor hetero-tetramer forms a transmembrane pore for the exchange of cations, such as Na^+^, K^+^ and Ca^2+^, across the membrane leading to membrane depolarization and facilitating action potential formation (Beaurain et al., 2024). Importantly, NMDA receptors are both voltage and ligand-gated receptors, with membrane depolarization necessary for the removal of the Mg^2+^ block to allow for the flow of ions across the membrane (Luo et al., 2011). Thus, early in development prior to AMPA receptor expression, NMDA receptors are thought to be non-functional or silent, although Ca^2+^ influx has been observed in the absence of AMPA and kainate receptor activity (Liao et al., 2001). Furthermore, recent evidence suggests that early in development NMDA receptors may negatively regulate AMPA receptor expression and function, allowing for developmental synaptic plasticity and preventing premature onset of synapse maturation (Hall and Ghosh, 2008). In the following review, we will address how developmentally regulated expression of specific GluN2 isoforms and their unique interactions with actin cytoskeletal regulators balances the competing events of synaptic plasticity and synaptic stabilization in developing neural circuits. We will also examine how alterations in isoform expression later in life can adversely impact synaptic stabilization, driving key events in neurodegeneration.

### Developmental expression of NMDA receptor subunits

**Figure 1:**
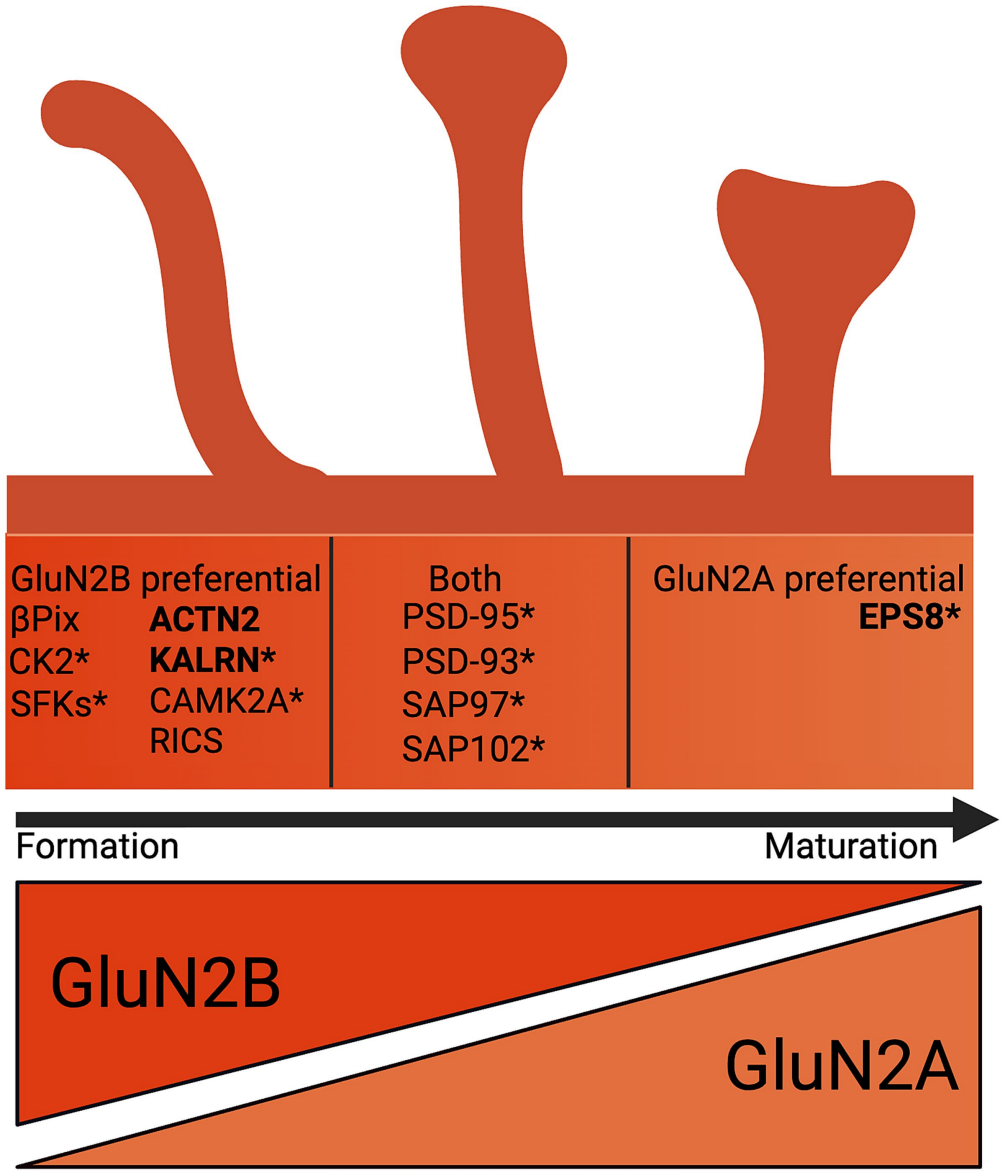
Diagram of NMDAR protein interactors known interaction networks of proteins associated with GluN2A or GluN2B-containing NMDARs. Genes indicated in bold exhibit predominant prenatal expression as determined by the Human Brain Transcriptome (Kang et al., 2011; Johnson et al., 2009). Documented direct interactions with Glun2 subunits are denoted by asterisks. Created in BioRender. Litwa, K. (2026). https://BioRender.com/gdets2hx.

NMDA receptor subunits exhibit unique spatial and temporal expression profiles in development. While one gene encodes the obligatory GluN1 subunit, four distinct genes encode unique regulatory GluN2 isoforms—GRIN2A, GRIN2B, GRIN2C, and GRIN2D (Myers et al., 2019). The obligate NMDA receptor subunit, GluN1, expresses early in embryonic development throughout the brain (Ewald et al., 2009). Similarly, GluN2B and GluN2D express earlier in the developing embryonic brain than the other GluN2 isoforms (Akazawa et al., 1994). GluN2B expression is widespread and particularly strong in the cortex, thalamus and spinal cord, while GluN2D expression is more restricted and is absent from the telencephalon (Ewald et al., 2009). GluN2B expression peaks in early post-natal development before declining to moderate levels in adulthood (Ladagu et al., 2023). In contrast to GluN2B, GluN2A expression initially increases in the hippocampus and cortex postnatally before it becomes dramatically upregulated throughout the brain by the second postnatal week (Ewald et al., 2009). GluN2C expression, predominantly in the cerebellum, also begins later in development around postnatal day 10 (Ewald et al., 2009; Akazawa et al., 1994; Ladagu et al., 2023; Paoletti et al., 2013). Thus, in the following review, we will compare the developmentally regulated expression of GluN2A and GluN2B (Figure 1), which are the most widespread GluN2 subunits in the brain and are expressed within cortical regions that regulate learning and memory formation. Furthermore, both mutations and/or altered expression of GluN2A/B drive the emergence of neurodevelopmental disorders, while also later contributing to neurodegeneration (Ball et al., 2020).

### NMDA receptor subunits confer unique ion channel properties

In addition to their unique expression profiles, NMDA receptor subunit structure underlies differential regulation of excitatory neurotransmission. Each NMDA receptor subunit consists of an extracellular N-terminal and ligand binding domain for D-serine/glycine in the case of GluN1 and glutamate for GluN2 isoforms, three transmembrane domains, a Mg^2+^-sensitive re-entrant loop which regulates ion permeability, and an intracellular C-terminal domain (CTD) (Traynelis et al., 2010). The predominantly expressed GluN2 isoforms, GluN2A and GluN2B, exhibit high homology in their extracellular and transmembrane domains (~70% identical), but relatively low homology (32% identical, 48% similar) in their intracellular CTD, which can facilitate unique intracellular protein interactions (Myers et al., 2019). However, even within the extracellular N-terminus, isoform-specific conformational differences and allosteric regulation confer unique kinetic properties to NMDAR ion channels (Tian et al., 2021). Notably, GluN2A-containing NMDARs exhibit faster synaptic currents, arising from enhanced glutamate sensitivity that increases the peak amplitude and open probability in response to glutamate and accelerates deactivation following glutamate removal when compared to GluN2B-containing NMDARs (Myers et al., 2019). These differences result in a stronger role for GluN2B in synaptic plasticity and strengthening, while GluN2A prevents further LTP-induced synaptic changes, and thereby stabilizes mature synaptic contacts (Shipton and Paulsen, 2014; Kellermayer et al., 2018; Gray et al., 2011).

### Actin regulation of spine dynamics

Synaptic plasticity and maturation are associated with distinct actin-driven spine dynamics and morphologies, thus highlighting the importance of coordinated NMDA receptor activity and actin dynamics for neural circuit formation and function (McKinney, 2010; Hlushchenko et al., 2016; Khanal and Hotulainen, 2021; Penzes et al., 2011; Hotulainen and Hoogenraad, 2010; Shaw and Koleske, 2021). In the cortex, spines emerge from the dendritic shaft as dynamic filopodia-like spine precursors with anti-parallel actin filaments (Hotulainen and Hoogenraad, 2010). Actin polymerization drives the emergence and elongation of spine precursors, while actin motor protein non-muscle myosin II (NMII) antagonizes spine precursor elongation (Hotulainen and Hoogenraad, 2010; Martin-Vilchez et al., 2017). With developmental maturation, a subset of spine precursors will transition to more stable polarized structures that exhibit a spine neck, still with anti-parallel actin filaments, and a bulbous spine head, with branched actin filaments (Hotulainen and Hoogenraad, 2010; Martin-Vilchez et al., 2017). In response to further NMDA receptor activation, spines shorten into mushroom-shaped structures with increased spine head size, increasing the post-synaptic density (PSD) surface area (Lynch et al., 2007; Park et al., 2004). The increased PSD surface area results in more NMDA and AMPA receptors adjacent to the pre-synaptic terminal, increasing the likelihood that the depolarization threshold will be met for action potential formation (Park et al., 2004). This phenomenon of activity dependent strengthening is referred to as long-term potentiation (LTP) (Lynch et al., 2007). In LTP, NMII promotes spine maturation (Hodges et al., 2011). In addition to NMII, other actin regulatory proteins coordinate these dynamic events associated with dendritic spine development. For example, in spine maturation, the actin severing protein cofilin initially severs actin filaments to initiate morphological expansion but is subsequently inactivated by phosphorylation of it Ser3 subunit, resulting in stabilization of the new actin arrangements underlying spine consolidation (Bosch et al., 2014). The actin nucleating protein, Arp2/3, forms branched actin arrays, underlying spine head expansion (Hotulainen and Hoogenraad, 2010). RhoGTPases regulate the activity of these actin-associated proteins. For example, the RhoGTPase family member, RhoA, through its effector kinase, ROCK1, activates NMII through phosphorylation of the regulatory light chain (Hodges et al., 2011; Newell-Litwa et al., 2015). In contrast, another RhoGTPase family member, Rac1, drives actin polymerization through Arp2/3 (Costa et al., 2020). There is also significant crosstalk between RhoGTPases, with Rac1 and RhoA exhibiting mutual antagonism (Salloum et al., 2020). For example, sequential activation of RhoA followed by Rac1 underlies LTP consolidation (Rex et al., 2009). This RhoGTPase activity is controlled by membrane-associated Guanine Exchange Factors (GEFs), which activate RhoGTPases through the exchange of GDP for GTP. GTPase-Activating Proteins (GAPs) stimulate GTP hydrolysis, resulting in RhoGTPase inactivation, which can be perpetuated by Guanine Dissociation Inhibitors (GDIs) which sequester GDP-bound RhoGTPases in the cytosol (Martin-Vilchez et al., 2017). Similar to GluN2 isoforms, RhoGTPase regulatory proteins exhibit differential expression during spine development and regulate distinct stages of spine precursor formation and spine maturation into mushroom-shaped spines (Martin-Vilchez et al., 2017). In the following sections, we will address how GluN2 isoforms associate with these intersecting actin regulatory pathways to coordinate spine structural and functional changes underlying learning and memory formation.

### GluN2A and Glun2B associations with the actin cytoskeleton coordinate activity-dependent spine development

Intracellularly, NMDA receptors bind to the Post-Synaptic Density (PSD) subfamily of synaptic scaffolding proteins, which include PSD-95, PSD-93, SAP102 and SAP97 (Shipton and Paulsen, 2014). These PSD proteins anchor to the underlying synaptic actin cytoskeleton through actin linking proteins, such as α-actinin (Matt et al., 2018). Interestingly isoform-specific post-translational modifications regulate GluN2 association with PSD binding partners, affecting their anchoring and expression at synapses (Shipton and Paulsen, 2014). For example, Casein Kinase-2 (CK2), which exhibits increased mRNA expression prenatally (Kang et al., 2011; Johnson et al., 2009), phosphorylates GluN2B at Ser1480 in response to NMDAR activation, disrupting its interaction with PSD-95 and SAP102 and leading to a reduction in synaptic GluN2B and a corresponding increase in GluN2A, thus facilitating the activity-dependent switch from synaptic plasticity to synapse maturation (Chung et al., 2004; Sanz-Clemente et al., 2010). Conversely, the Src Family Kinases (SFKs) phosphorylate GluN2B at Tyr1472, preventing AP-2 binding and endocytosis, and thereby increase GluN2B surface expression (Zhang et al., 2008). In contrast to the phospho-regulated internalization of GluN2B through its YEKL endocytic sorting motif, GluN2A endocytosis is regulated by a di-leucine sorting motif (Shipton and Paulsen, 2014), reflecting independent mechanisms governing surface expression and scaffold association of the two subunits.

In addition to their association with post-synaptic scaffold proteins, GluN2 subunits exhibit preferential interactions with actin regulatory proteins. The early developmental expression of GluN2B uniquely positions it as a gatekeeper of subsequent synaptic maturation. Within post-synaptic scaffolds, GluN2B regulates actin cytoskeletal rearrangements through its interaction with the RhoGAP, p250GAP (also known as ArhGAP32 and RICS), a negative regulator of RhoA, Rac1, and Cdc42 activity (Shaw and Koleske, 2021; Nakazawa et al., 2003, 2008; Okabe et al., 2003). Importantly, in response to NMDA receptor activation, CAMKII [which also binds to GluN2B (Curtis et al., 2023)], phosphorylates and inactivates p250GAP, allowing for sequential RhoGTPase activation necessary for LTP consolidation (Rex et al., 2009; Okabe et al., 2003; Nakazawa et al., 2008). In addition to p250GAP-mediated regulation of spine maturation, GluN2B associates with the actin crosslinking protein, α-actinin-2, which bundles antiparallel actin filaments to initiate spine maturation (Nakazawa et al., 2003; Okabe et al., 2003; Nakazawa et al., 2008; Curtis et al., 2023; Hodges et al., 2014; Hu et al., 2016; Wyszynski et al., 1997). This engagement with α-actinin-2 is also activity-regulated. In response to NMDA receptor activation, GluN2B associates with the substrate binding groove of CaMKII, promoting the interaction between CaMKII and α-actinin-2 and resulting in actin crosslinking and spine maturation (Curtis et al., 2023). As demonstrated by the above examples, GluN2B binding to CaMKII, critically regulates LTP (Incontro et al., 2018), and likely leads to engagement with other GTPase regulatory proteins which have yet to be fully elucidated (Shibata et al., 2021). GluN2B’s pivotal role as an initiator of synaptic maturation is further demonstrated by conditional GluN2B knockout studies, which result in reduced dendritic spines, despite sustained GluN2A expression (Akashi et al., 2009). While GluN2B association with CAMKII initiates spine maturation through regulation of the actin cytoskeleton, the pathways by which GluN2B regulates dynamic actin polymerization associated with filopodia-like spine precursors is less clear. However, while these pathways are not fully elucidated, GluN2B is known to interact with the Rac1 GEF, Kalirin (Kiraly et al., 2011), which promotes actin polymerization. Furthermore, the Rac GEF, β-Pix, which is associated with spine precursor formation (Martin-Vilchez et al., 2017), tightly regulates GluN2B expression (Kwon et al., 2025). Thus, GluN2B association with Rac-GEFs likely promotes spine precursor formation and elongation, but subsequent NMDA receptor activation drives association with CaMKII and results in actin rearrangements through actin-binding proteins, such as α-actinin-2, that drive the morphological maturation into mushroom-shaped spines (El Gaamouch et al., 2012).

In contrast to GluN2B, GluN2A interacts more strongly with post-synaptic scaffolds and does not undergo lateral diffusion during LTP (Dupuis et al., 2014). Actin stabilization enhances Glun2A association with synaptic scaffolds. For example, Eps8 is an actin capping protein which binds to GluN2A and stabilizes actin filaments (Morini et al., 2018). Conversely, phosphorylation of Eps8 inhibits its actin capping function and stimulates filipodia extension (Menna et al., 2009). In mature hippocampal neurons, Eps8 is recruited to the spine head during LTP and is associated with spine enlargement. Eps8 knockout reduced synaptic GluN2A expression, while resulting in a corresponding increase in GluN2B expression (Morini et al., 2018). Thus, while GluN2B regulates dynamic actin rearrangements, Glun2A favors association with stabilized actin filaments, thereby preventing further spine plasticity. Thus, unlike GluN2B, which regulates dynamic actin rearrangements underlying spine plasticity and strengthening, GluN2A contributes to stabilization of synaptic contacts.

### GluN2A and GluN2B disruptions contribute to neurological disorders

Multiple neurodevelopmental disorders are associated with the GluN2 subunits, with GluN2B mutations commonly resulting in neurodevelopmental disorders while GluN2A mutations more commonly lead to epilepsy (Myers et al., 2019). The GRIN2B gene exhibits over 200 different variants in patients with several neurological conditions including intellectual disability, developmental delay, autism spectrum disorder, schizophrenia, and attention deficit/hyperactivity disorder, highlighting GluN2B’s critical regulation of early developmental synaptic plasticity (Myers et al., 2019). However, consistent with regulation of synapse maturation, GluN2A mutations commonly result in epilepsy, such as benign focal epilepsy with centrotemporal spikes (BECTS) (Myers et al., 2019). Differences in the ratio of GluN2A to GluN2B can also contribute to neurodevelopmental disorders. For example, Fragile X Syndrome results in increased GluN2A expression (Banke et al., 2024), which impairs actin-driven synaptic plasticity, but can be rescued by swapping the Glun2A CTD with the GluN2B CTD, allowing for regulation of actin dynamics (Barnes et al., 2025). Furthermore, while GluN2B expression is highest during early development, further age-related changes in GluN2B expression are associated with neurodegenerative diseases, such as Alzheimer’s Diease (Andreoli et al., 2013; Martel et al., 2012; Xu et al., 2023). Both increased and decreased GluN2B expression have been implicated in Alzheimer’s Disease pathogenesis. Decreased GluN2B expression impairs synaptic plasticity (Andreoli et al., 2013), and in a mouse model of Alzheimer’s Disease, GluN2B expression rescued mice from cognitive decline (Brim et al., 2013). However, elevated GluN2B expression can also contribute to Alzheimer’s Disease through elevated excitotoxicity and cell death (Martel et al., 2012; Xu et al., 2023). Under these conditions, the NMDA receptor antagonist, memantine, exhibits some therapeutic efficacy by preventing further neuronal cell loss (Limapichat et al., 2013).

## Discussion

In this review, we have highlighted how GluN2B and GluN2A coordinate dendritic spine development through regulation of the actin cytoskeleton. GluN2B regulates dynamic actin rearrangements associated with synaptic plasticity and synaptic strengthening. GluN2B expression begins early in development, when immature filopodia-like spine precursors predominate. These filopodia-like spine precursors exhibit rapid actin-driven elongation and retraction. Consistent with its early developmental expression, Glun2B associates with signaling pathways that promote actin polymerization, such as those driven by the Rac GEFs, β-Pix and Kalirin, which also exhibit elevated expression early in development (Figure 1) (Kiraly et al., 2011; Kwon et al., 2025). However, while GluN2B expression peaks in early post-natal development, it remains expressed throughout life and promotes synaptic strengthening by regulating the morphological maturation to mushroom-shaped spines. In response to NMDA receptor activation, CaMKII negatively regulates GluN2B association with the RhoGAP, RICS, allowing for sequential RhoGTPase activation necessary for activity-driven spine maturation (Okabe et al., 2003). CaMKII also promotes GluN2B association with α-actinin-2 to crosslink actin filaments, supporting the polarized morphology of dendritic spines (Hodges et al., 2014; Wyszynski et al., 1997). In contrast, Glun2A more stably associates with post-synaptic scaffolds and interacts with stable actin arrangements, as highlighted by its association with the actin capping protein, Eps8 (Menna et al., 2009). This association prevents further spine morphology changes. Not surprisingly, changes in Glun2A and GluN2B expression disrupt actin-driven spine development, resulting in neurodevelopmental disorders and contributing to neurodegenerative diseases later in life (Myers et al., 2019). Further identification of GluN2 isoform-specific actin interactions at specific stages of development will aid in targeted development of therapeutics for these disorders.

## References

[ref1] Akashi K. Kakizaki T. Kamiya H. Fukaya M. Yamasaki M. Abe M. . (2009). NMDA receptor GluN2B (GluR epsilon 2/NR2B) subunit is crucial for channel function, postsynaptic macromolecular organization, and actin cytoskeleton at hippocampal CA3 synapses. J. Neurosci. 29, 10869–10882. doi: 10.1523/JNEUROSCI.5531-08.2009, 19726645 PMC6665524

[ref2] Akazawa C. Shigemoto R. Bessho Y. Nakanishi S. Mizuno N. (1994). Differential expression of five N-methyl-D-aspartate receptor subunit mRNAs in the cerebellum of developing and adult rats. J. Comp. Neurol. 347, 150–160. doi: 10.1002/cne.903470112, 7798379

[ref3] Andreoli V. de Marco E. V. Trecroci F. Cittadella R. di Palma G. Gambardella A. (2013). Potential involvement of GRIN2B encoding the NMDA receptor subunit NR2B in the spectrum of Alzheimer’s disease. J. Neural Transm. 121, 533–542. doi: 10.1007/s00702-013-1125-7 24292895

[ref4] Ball G. Seidlitz J. Beare R. Seal M. L. (2020). Cortical remodelling in childhood is associated with genes enriched for neurodevelopmental disorders. NeuroImage 215:116803. doi: 10.1016/j.neuroimage.2020.116803, 32276068

[ref1001] Banke T. G. Traynelis S. F. Barria A. (2024). Early expression of GluN2A-containing NMDA receptors in a model of fragile X syndrome. J. Neurophysiol. 131, 768–777. doi: 10.1152/jn.00406.2023, 38380828 PMC11254340

[ref5] Barnes S. A. Thomazeau A. Finnie P. S. B. Heinrich M. J. Heynen A. J. Komiyama N. H. . (2025). Non-ionotropic signaling through the NMDA receptor GluN2B carboxy-terminal domain drives dendritic spine plasticity and reverses fragile X phenotypes. Cell Rep. 44:115311. doi: 10.1016/j.celrep.2025.115311, 39983718 PMC12006837

[ref6] Beaurain M. Salabert A.-S. Payoux P. Gras E. Talmont F. (2024). NMDA receptors: distribution, role, and insights into neuropsychiatric disorders. Pharmaceuticals 17:1265. doi: 10.3390/ph17101265, 39458906 PMC11509972

[ref7] Bosch M. Castro J. Saneyoshi T. Matsuno H. Sur M. Hayashi Y. (2014). Structural and molecular remodeling of dendritic spine substructures during long-term potentiation. Neuron 82, 444–459. doi: 10.1016/j.neuron.2014.03.021, 24742465 PMC4281348

[ref8] Brim B. L. Haskell R. Awedikian R. Ellinwood N. M. Jin L. Kumar A. . (2013). Memory in aged mice is rescued by enhanced expression of the GluN2B subunit of the NMDA receptor. Behav. Brain Res. 238, 211–226. doi: 10.1016/j.bbr.2012.10.026, 23103326 PMC3540206

[ref9] Chung H. J. Huang Y. H. Lau L.-F. Huganir R. L. (2004). Regulation of the NMDA receptor complex and trafficking by activity-dependent phosphorylation of the NR2B subunit PDZ ligand. J. Neurosci. 24, 10248–10259. doi: 10.1523/JNEUROSCI.0546-04.2004, 15537897 PMC6730169

[ref10] Costa J. F. Dines M. Lamprecht R. (2020). The role of Rac GTPase in dendritic spine morphogenesis and memory. Front Synaptic Neurosci 12:12. doi: 10.3389/fnsyn.2020.00012, 32362820 PMC7182350

[ref11] Curtis A. J. Zhu J. Penny C. J. Gold M. G. (2023). Molecular basis of interactions between CaMKII and α-actinin-2 that underlie dendritic spine enlargement. eLife 12:e85008. doi: 10.7554/eLife.85008, 37489746 PMC10484527

[ref12] Dupuis J. P. Ladepeche L. Seth H. Bard L. Varela J. Mikasova L. . (2014). Surface dynamics of GluN2B-NMDA receptors controls plasticity of maturing glutamate synapses. EMBO J. 33, 842–861. doi: 10.1002/embj.201386356, 24591565 PMC4194110

[ref13] El Gaamouch F. Buisson A. Moustié O. Lemieux M. Labrecque S. Bontempi B. . (2012). Interaction between αCaMKII and GluN2B controls ERK-dependent plasticity. J. Neurosci. 32:22855824, 10767–10779. doi: 10.1523/JNEUROSCI.5622-11.2012 PMC6621385 22855824

[ref43] Ewald R. C. Cline H. T. (2009). “NMDA Receptors and Brain Development.” in Biology of the NMDA Receptor, Chapter 1. ed. A. M. Van Dongen (Boca Raton, FL: CRC Press/Taylor & Francis). 21204419

[ref14] Gray J. A. Shi Y. Usui H. During M. J. Sakimura K. Nicoll R. A. (2011). Distinct modes of AMPA receptor suppression at developing synapses by GluN2A and GluN2B: single-cell NMDA receptor subunit deletion in vivo. Neuron 71, 1085–1101. doi: 10.1016/j.neuron.2011.08.007, 21943605 PMC3183990

[ref15] Hall B. J. Ghosh A. (2008). Regulation of AMPA receptor recruitment at developing synapses. Trends Neurosci. 31, 82–89. doi: 10.1016/j.tins.2007.11.010, 18201773

[ref16] Hlushchenko I. Koskinen M. Hotulainen P. (2016). Dendritic spine actin dynamics in neuronal maturation and synaptic plasticity. Cytoskeleton 73, 435–441. doi: 10.1002/cm.21280, 26849484

[ref17] Hodges J. L. Newell-Litwa K. Asmussen H. Vicente-Manzanares M. Horwitz A. R. (2011). Myosin IIB activity and phosphorylation status determines dendritic spine and post-synaptic density morphology. PLoS One 6:e24149. doi: 10.1371/journal.pone.0024149, 21887379 PMC3162601

[ref18] Hodges J. L. Vilchez S. M. Asmussen H. Whitmore L. A. Horwitz A. R. (2014). α-Actinin-2 mediates spine morphology and assembly of the post-synaptic density in hippocampal neurons. PLoS One 9:e101770. doi: 10.1371/journal.pone.0101770, 25007055 PMC4090192

[ref19] Hotulainen P. Hoogenraad C. C. (2010). Actin in dendritic spines: connecting dynamics to function. J. Cell Biol. 189, 619–629. doi: 10.1083/jcb.201003008, 20457765 PMC2872912

[ref20] Hu C. Chen W. Myers S. J. Yuan H. Traynelis S. F. (2016). Human GRIN2B variants in neurodevelopmental disorders. J. Pharmacol. Sci. 132, 115–121. doi: 10.1016/j.jphs.2016.10.002, 27818011 PMC5125235

[ref21] Incontro S. Díaz-Alonso J. Iafrati J. Vieira M. Asensio C. S. Sohal V. S. . (2018). The CaMKII/NMDA receptor complex controls hippocampal synaptic transmission by kinase-dependent and independent mechanisms. Nat. Commun. 9:2069. doi: 10.1038/s41467-018-04439-7, 29802289 PMC5970233

[ref22] Johnson M. B. Kawasawa Y. I. Mason C. E. Krsnik Ž. Coppola G. Bogdanović D. . (2009). Functional and evolutionary insights into human brain development through global transcriptome analysis. Neuron 62, 494–509. doi: 10.1016/j.neuron.2009.03.027, 19477152 PMC2739738

[ref23] Kang H. J. Kawasawa Y. I. Cheng F. Zhu Y. Xu X. Li M. . (2011). Spatio-temporal transcriptome of the human brain. Nature 478, 483–489. doi: 10.1038/nature10523, 22031440 PMC3566780

[ref24] Kellermayer B. Ferreira J. S. Dupuis J. Levet F. Grillo-Bosch D. Bard L. . (2018). Differential nanoscale topography and functional role of GluN2-NMDA receptor subtypes at glutamatergic synapses. Neuron 100, 106–119.e7. doi: 10.1016/j.neuron.2018.09.012, 30269991

[ref25] Khanal P. Hotulainen P. (2021). Dendritic spine initiation in brain development, learning and diseases and impact of BAR-domain proteins. Cells 10:2392. doi: 10.3390/cells10092392, 34572042 PMC8468246

[ref26] Kiraly D. D. Lemtiri-Chlieh F. Levine E. S. Mains R. E. Eipper B. A. (2011). Kalirin binds the NR2B subunit of the NMDA receptor, altering its synaptic localization and function. J. Neurosci. 31, 12554–12565. doi: 10.1523/JNEUROSCI.3143-11.2011, 21880917 PMC3172699

[ref27] Kwon Y. Lee S. J. Shin Y. K. Choi J. S. Park D. Shin J. E. (2025). Loss of neuronal βPix isoforms impairs neuronal morphology in the hippocampus and causes behavioral defects. Anim Cells Syst (Seoul) 29, 57–71. doi: 10.1080/19768354.2024.2448999, 39802101 PMC11722029

[ref28] Ladagu A. D. Olopade F. E. Adejare A. Olopade J. O. (2023). GluN2A and GluN2B N-methyl-D-aspartate receptor (NMDARs) subunits: their roles and therapeutic antagonists in neurological diseases. Pharmaceuticals 16:1535. doi: 10.3390/ph16111535, 38004401 PMC10674917

[ref29] Liao D. Scannevin R. H. Huganir R. (2001). Activation of silent synapses by rapid activity-dependent synaptic recruitment of AMPA receptors. J. Neurosci. 21, 6008–6017. doi: 10.1523/JNEUROSCI.21-16-06008.2001, 11487624 PMC6763128

[ref30] Limapichat W. Yu W. Y. Branigan E. Lester H. A. Dougherty D. A. (2013). Key binding interactions for memantine in the NMDA receptor. ACS Chem. Neurosci. 4, 255–260. doi: 10.1021/cn300180a, 23421676 PMC3751542

[ref31] Luo T. Wu W.-H. Chen B.-S. (2011). NMDA receptor signaling: death or survival? Front. Biol. 6, 468–476. doi: 10.1007/s11515-011-1187-6, 23144645 PMC3491906

[ref32] Lynch G. Rex C. S. Gall C. M. (2007). LTP consolidation: substrates, explanatory power, and functional significance. Neuropharmacology 52, 12–23. doi: 10.1016/j.neuropharm.2006.07.027, 16949110

[ref33] Martel M.-A. Ryan T. J. Bell K. F. S. Fowler J. H. McMahon A. al-Mubarak B. . (2012). The subtype of GluN2 C-terminal domain determines the response to excitotoxic insults. Neuron 74, 543–556. doi: 10.1016/j.neuron.2012.03.021, 22578505 PMC3398391

[ref34] Martin-Vilchez S. Whitmore L. Asmussen H. Zareno J. Horwitz R. Newell-Litwa K. (2017). RhoGTPase regulators orchestrate distinct stages of synaptic development. PLoS One 12:e0170464. doi: 10.1371/journal.pone.0170464, 28114311 PMC5256999

[ref35] Matt L. Kim K. Hergarden A. C. Patriarchi T. Malik Z. A. Park D. K. . (2018). α-Actinin anchors PSD-95 at postsynaptic sites. Neuron 97, 1094–1109.e9. doi: 10.1016/j.neuron.2018.01.036, 29429936 PMC5963734

[ref36] McKinney R. A. (2010). Excitatory amino acid involvement in dendritic spine formation, maintenance and remodelling. J. Physiol. 588, 107–116. doi: 10.1113/jphysiol.2009.178905, 19933758 PMC2821552

[ref37] Menna E. Disanza A. Cagnoli C. Schenk U. Gelsomino G. Frittoli E. . (2009). Eps8 regulates axonal filopodia in hippocampal neurons in response to brain-derived neurotrophic factor (BDNF). PLoS Biol. 7:e1000138. doi: 10.1371/journal.pbio.1000138, 19564905 PMC2696597

[ref38] Morini R. Ferrara S. Perrucci F. Zambetti S. Pelucchi S. Marcello E. . (2018). Lack of the actin capping protein, Eps8, affects NMDA-type glutamate receptor function and composition. Front. Mol. Neurosci. 11:313. doi: 10.3389/fnmol.2018.00313, 30233314 PMC6133960

[ref39] Myers S. J. Yuan H. Kang J. Q. Tan F. C. K. Traynelis S. F. Low C. M. (2019). Distinct roles of GRIN2A and GRIN2B variants in neurological conditions. F1000Res 8:1940. doi: 10.12688/f1000research.18949.1 PMC6871362 31807283

[ref40] Nakazawa T. Kuriu T. Tezuka T. Umemori H. Okabe S. Yamamoto T. (2008). Regulation of dendritic spine morphology by an NMDA receptor-associated rho GTPase-activating protein, p250GAP. J. Neurochem. 105, 1384–1393. doi: 10.1111/j.1471-4159.2008.05335.x, 18331582

[ref41] Nakazawa T. Watabe A. M. Tezuka T. Yoshida Y. Yokoyama K. Umemori H. . (2003). p250GAP, a novel brain-enriched GTPase-activating protein for rho family GTPases, is involved in the N-methyl-d-aspartate receptor signaling. Mol. Biol. Cell 14, 2921–2934. doi: 10.1091/mbc.e02-09-0623, 12857875 PMC165687

[ref42] Newell-Litwa K. A. Badoual M. Asmussen H. Patel H. Whitmore L. Horwitz A. R. (2015). ROCK1 and 2 differentially regulate actomyosin organization to drive cell and synaptic polarity. J. Cell Biol. 210, 225–242. doi: 10.1083/jcb.201504046, 26169356 PMC4508895

[ref44] Okabe T. Nakamura T. Nishimura Y. N. Kohu K. Ohwada S. Morishita Y. . (2003). RICS, a novel GTPase-activating protein for Cdc42 and Rac1, is involved in the β-catenin-N-cadherin andN-methyl-d-aspartate receptor signaling. J. Biol. Chem. 278, 9920–9927. doi: 10.1074/jbc.M208872200, 12531901

[ref45] Paoletti P. Bellone C. Zhou Q. (2013). NMDA receptor subunit diversity: impact on receptor properties, synaptic plasticity and disease. Nat. Rev. Neurosci. 14, 383–400. doi: 10.1038/nrn3504, 23686171

[ref46] Park M. Penick E. C. Edwards J. G. Kauer J. A. Ehlers M. D. (2004). Recycling endosomes supply AMPA receptors for LTP. Science 305, 1972–1975. doi: 10.1126/science.1102026, 15448273

[ref47] Penzes P. Cahill M. E. Jones K. A. VanLeeuwen J.-E. Woolfrey K. M. (2011). Dendritic spine pathology in neuropsychiatric disorders. Nat. Neurosci. 14, 285–293. doi: 10.1038/nn.2741, 21346746 PMC3530413

[ref48] Rex C. S. Chen L. Y. Sharma A. Liu J. Babayan A. H. Gall C. M. . (2009). Different rho GTPase–dependent signaling pathways initiate sequential steps in the consolidation of long-term potentiation. J. Cell Biol. 186, 85–97. doi: 10.1083/jcb.200901084, 19596849 PMC2712993

[ref49] Salloum G. Jaafar L. El-Sibai M. (2020). Rho a and Rac1: antagonists moving forward. Tissue Cell 65:101364. doi: 10.1016/j.tice.2020.101364, 32746999

[ref50] Sanz-Clemente A. Matta J. A. Isaac J. T. R. Roche K. W. (2010). Casein kinase 2 regulates the NR2 subunit composition of synaptic NMDA receptors. Neuron 67, 984–996. doi: 10.1016/j.neuron.2010.08.011, 20869595 PMC2947143

[ref51] Shaw J. E. Koleske A. J. (2021). Functional interactions of ion channels with the actin cytoskeleton: does coupling to dynamic actin regulate NMDA receptors? J. Physiol. 599, 431–441. doi: 10.1113/JP278702, 32034761 PMC7416480

[ref52] Shibata A. C. E. Ueda H. H. Eto K. Onda M. Sato A. Ohba T. . (2021). Photoactivatable CaMKII induces synaptic plasticity in single synapses. Nat. Commun. 12:751. doi: 10.1038/s41467-021-21025-6, 33531495 PMC7854602

[ref53] Shipton O. A. Paulsen O. (2014). GluN2A and GluN2B subunit-containing NMDA receptors in hippocampal plasticity. Philos. Trans. R. Soc. B 369:20130163. doi: 10.1098/rstb.2013.0163, 24298164 PMC3843894

[ref54] Tian M. Stroebel D. Piot L. David M. Ye S. Paoletti P. (2021). GluN2A and GluN2B NMDA receptors use distinct allosteric routes. Nat. Commun. 12:4709. doi: 10.1038/s41467-021-25058-9, 34354080 PMC8342458

[ref55] Traynelis S. F. Wollmuth L. P. McBain C. J. Menniti F. S. Vance K. M. Ogden K. K. . (2010). Glutamate receptor ion channels: structure, regulation, and function. Pharmacol. Rev. 62, 405–496. doi: 10.1124/pr.109.002451, 20716669 PMC2964903

[ref56] Wyszynski M. Lin J. Rao A. Nigh E. Beggs A. H. Craig M. (1997). Competitive binding of α-actinin and calmodulin to the NMDA receptor. Nature 385, 439–442. doi: 10.1038/385439a0 9009191

[ref57] Xu L.-Z. Li B. Q. Li F. Y. Li Y. Qin W. Zhao Y. . (2023). NMDA receptor GluN2B subunit is involved in excitotoxicity mediated by death-associated protein kinase 1 in Alzheimer’s disease. J. Alzheimer's Dis 91, 877–893. doi: 10.3233/JAD-220747, 36502323

[ref58] Zhang S. Edelmann L. Liu J. Crandall J. E. Morabito M. A. (2008). Cdk5 regulates the phosphorylation of tyrosine 1472 NR2B and the surface expression of NMDA receptors. J. Neurosci. 28, 415–424. doi: 10.1523/JNEUROSCI.1900-07.2008, 18184784 PMC6670547

